# Accurate prediction of protein-protein interactions by integrating potential evolutionary information embedded in PSSM profile and discriminative vector machine classifier

**DOI:** 10.18632/oncotarget.15564

**Published:** 2017-02-21

**Authors:** Zheng-Wei Li, Zhu-Hong You, Xing Chen, Li-Ping Li, De-Shuang Huang, Gui-Ying Yan, Ru Nie, Yu-An Huang

**Affiliations:** ^1^ School of Computer Science and Technology, China University of Mining and Technology, Xuzhou 221116, China; ^2^ Xinjiang Technical Institute of Physics and Chemistry, Chinese Academy of Science, Urumqi 830011, China; ^3^ School of Information and Control Engineering, China University of Mining and Technology, Xuzhou 221116, China; ^4^ School of Electronics and Information Engineering, Tongji University, Shanghai 201804, China; ^5^ Academy of Mathematics and Systems Science, Chinese Academy of Sciences, Beijing 100190, China; ^6^ College of Computer Science and Software Engineering, Shenzhen University, Shenzhen, Guangdong 518060, China

**Keywords:** disease, position-specific scoring matrix, Weber Local Descriptor, cancer, protein-protein interactions

## Abstract

Identification of protein-protein interactions (PPIs) is of critical importance for deciphering the underlying mechanisms of almost all biological processes of cell and providing great insight into the study of human disease. Although much effort has been devoted to identifying PPIs from various organisms, existing high-throughput biological techniques are time-consuming, expensive, and have high false positive and negative results. Thus it is highly urgent to develop *in silico* methods to predict PPIs efficiently and accurately in this post genomic era. In this article, we report a novel computational model combining our newly developed discriminative vector machine classifier (DVM) and an improved Weber local descriptor (IWLD) for the prediction of PPIs. Two components, differential excitation and orientation, are exploited to build evolutionary features for each protein sequence. The main characteristics of the proposed method lies in introducing an effective feature descriptor IWLD which can capture highly discriminative evolutionary information from position-specific scoring matrixes (PSSM) of protein data, and employing the powerful and robust DVM classifier. When applying the proposed method to *Yeast* and *H*. *pylori* data sets, we obtained excellent prediction accuracies as high as 96.52% and 91.80%, respectively, which are significantly better than the previous methods. Extensive experiments were then performed for predicting cross-species PPIs and the predictive results were also pretty promising. To further validate the performance of the proposed method, we compared it with the state-of-the-art support vector machine (SVM) classifier on *Human* data set. The experimental results obtained indicate that our method is highly effective for PPIs prediction and can be taken as a supplementary tool for future proteomics research.

## INTRODUCTION

In this post-genomic era, protein-protein interactions (PPIs) can provide great insights into the intrinsic mechanisms of biological processes within a cell and so the PPI networks have been drawing increasing attention. Recently, a number of high-throughput biological techniques, such as yeast two hybrid screens [1], mass spectrometric protein complex identification (MS-PCI) [2] and protein chips [3], have been proposed to identify interactions between proteins. Therefore, a large amount of PPI data from various kinds of organisms has been collected, and a number of databases, like DIP [4], BIND [5] and MINT [6], have also been constructed. However, such experimental methods for identifying PPIs are usually labor-intensive and time-consuming. The PPI pairs identified by these traditional techniques only account for a small part of the entire PPIs network [7, 8]. What's worse, those high-throughput techniques suffer from high rates of false positive and false negative results. All these limitations require robust and effective in silico methods as a complement to biological experimental techniques for protein-protein interactions prediction.

As a beneficial supplement to biological methods, a number of computational methods have been developed to predict protein interactions through different source of information, such as protein domains, phylogenetic profiles, gene co-expression and secondary structures [9–12]. However, such methods need specific domain knowledge which prevents their further applications. Evolutionary information embedded in proteins sequence has good capability for predicting PPIs [13]. Zahiri *et al*. [14] proposed a novel algorithm named PPIevo for detecting PPIs, which extracted the evolutionary feature from position-specific scoring matrixes (PSSM) of protein sequence. Hamp *et al*. [15] combined evolutionary profiles from protein sequence with profile-kernel support vector machines (SVM) to predict PPIs and obtained good results. An *et al*. [16] reported RVM-BiGP prediction model to predict PPIs from protein sequences and the results are very promising. Nevertheless, there is still room to improve the performance of the state-of-the-art prediction methods.

This paper is an extension of our previous work [17]. In this study, we report a novel computational model to predict PPIs using the evolutionary information of protein. The main improvements of the proposed method lie in introducing an effective feature extraction method, namely improved Weber local descriptor (IWLD) and using our newly developed discriminative vector machine (DVM) classifier. Specifically, given a protein sequence of length L, it would first be converted to an L-by-20 position-specific scoring matrix (PSSM). Then, an IWLD descriptor is used to extract discriminative evolutionary information from PSSM and a 256-dimensional histogram feature vector for each protein is constructed accordingly. Next, we combined two histogram vectors from corresponding protein pair into a 512-dimensional feature vector. Furthermore, the dimensionality reduction tool PCA (principal component analysis) is employed to extract the highly discriminatory information and reduce noise information. At last, the DVM classifier is used to carry out classification prediction. In this work, we first evaluated the proposed method on two PPIs data sets, *Yeast* and *H. pylori* and obtained good predictive accuracies of 96.52% and 91.80% respectively. Then, extensive experiments were performed to compare the proposed method with the state-of-the-art SVM classifier based on *Human* data set. Besides, comparisons between our method and other previous methods were also carried out. All the experimental results obtained indicate that the proposed method is impressively effective for PPIs prediction.

## RESULTS AND DISCUSSION

### Evaluation of predictive ability

To decrease data dependence and avoid over-fitting of prediction model, five-fold cross validation strategy was used in our study. Namely, the whole data set was evenly divided into five subsets, four of which were randomly chosen for training, and the rest for testing. To validate the validity of the proposed method, the random selection was repeated for five times, and five training sets and five validation sets were generated respectively. To be fair, parameters of DVM in different experiments were set to the same values. The predictive results of the proposed method on *Yeast* and *H. pylori* PPIs data sets are shown in Table 1 and Table 2.

**Table 1:** Performance of the proposed method using five-fold cross validation on Yeast data set

Test set	Acc (%)	Sen (%)	Pre (%)	MCC (%)
**1**	95.89	94.06	97.82	91.85
**2**	96.16	94.41	97.63	92.35
**3**	96.87	95.23	98.65	93.80
**4**	96.92	95.14	98.60	93.89
**5**	96.74	95.44	97.85	93.50
**Average**	**96.52±0.46**	**94.86±0.59**	**98.11±0.48**	**93.08±0.92**

**Table 2:** Performance of the proposed method using five-fold cross validation on *H. Pylori* data set

Test set	Acc (%)	Sen (%)	Pre (%)	MCC (%)
**1**	92.62	93.25	92.95	85.18
**2**	91.08	89.96	91.27	82.13
**3**	92.11	93.15	91.28	84.24
**4**	90.74	91.07	90.44	81.48
**5**	92.47	93.33	91.41	84.95
**Average**	**91.80±0.85**	**92.15±1.54**	**91.47±0.91**	**83.60±1.69**

**Figure 1:**
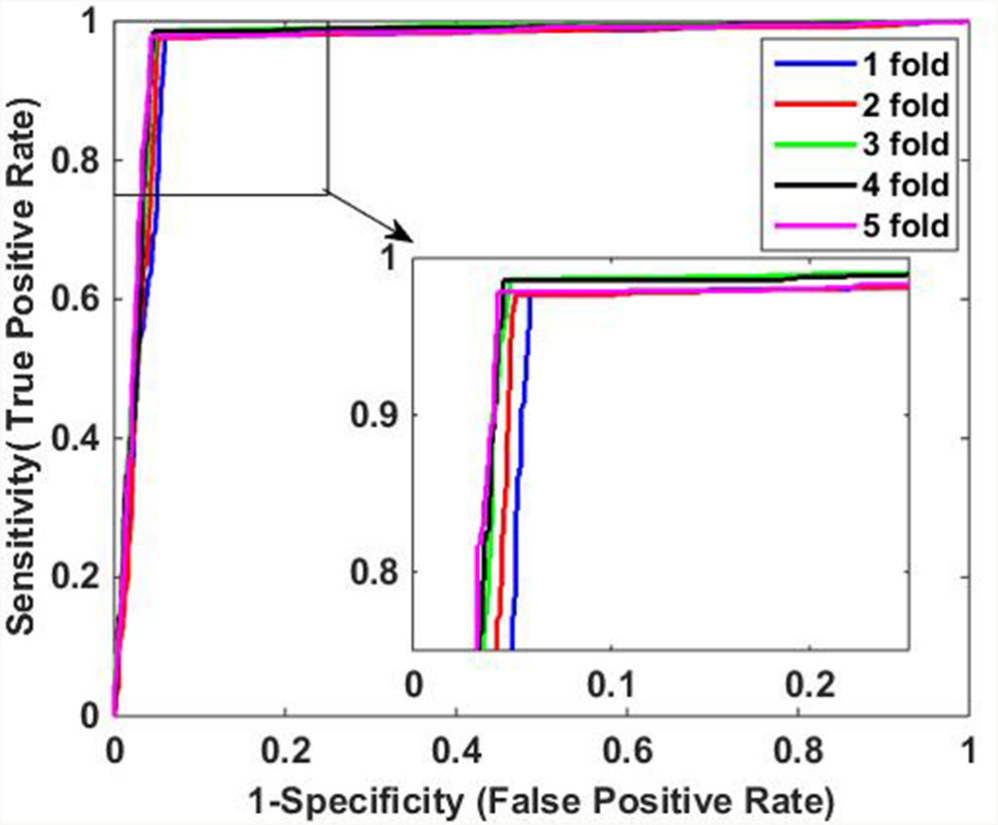
ROC curves of proposed method on *Yeast* data set

**Figure 2:**
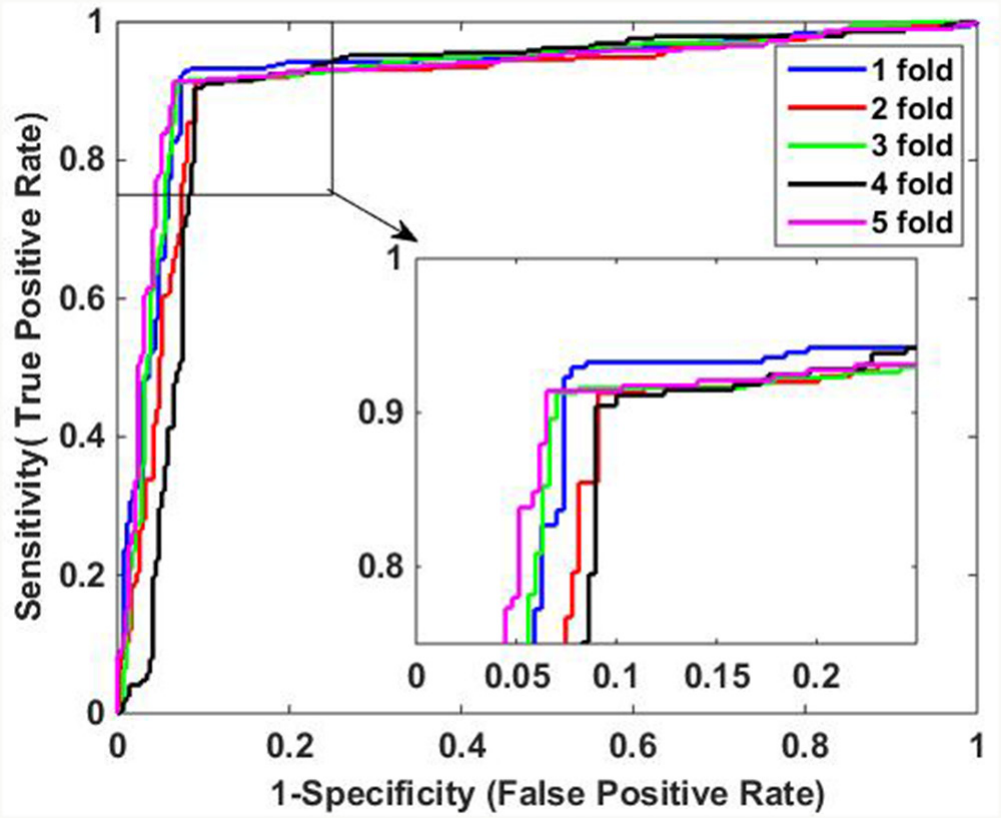
ROC curves of proposed method on *H. Pylori* data set

It can be observed from Table 1 that when applied to *Yeast* data set, the average accuracy, sensitivity, precision and MCC of the proposed method are 96.52%, 94.86%, 98.11%, and 93.08%, respectively. Similarly, Table 2 shows the results on *H. pylori* data set, it can be observed that the average accuracy obtained using our method is 91.80%, with an average sensitivity of 92.15%, an average precision of 91.47%, and an average MCC of 83.60%. In addition, it can be noticed that the standard deviations of them are also relatively low. For *Yeast* data set, the average standard deviations of accuracy, sensitivity, precision and MCC are 0.46%, 0.59%, 0.48% and 0.92%, respectively. The average standard deviations of accuracy, sensitivity, precision and MCC on *H. pylori* data set are 0.85%, 1.54%, 0.91% and 1.69%, respectively. The ROC curves using five-fold cross-validation on *Yeast* and *H. pylori* data sets are illustrated in Figure 1 and Figure 2, respectively.

From Table 1 and Table 2, it can be drawn that the proposed predictive model combing DVM and IWLD descriptor is accurate and effective for the prediction of PPIs from the two data sets. In our predictive model, PSSM not only provides the order information of protein sequence but also retains sufficient evolutionary information. Next, by using differential excitation and orientation component, the IWLD descriptor has strong ability to maintain local highly discriminative information for PPIs prediction. Besides, the application of PCA reduces the dimensions of IWLD vector, decreases the impact of noise and accelerates the predictive process. Consequently, our proposed method is suitable for predicting PPIs from the two data sets.

### Comparison with SVM classification model

Support vector machine (SVM) is one of the most widely used classification models for PPIs prediction. In this study, we used LIBSVM toolbox to carry out the prediction of PPIs (available at http://www.csie.ntu.edu.tw/~cjlin/libsvm/). To further verify the performance of the proposed method, we applied SVM to predict PPIs of *Human* data set and compared its performance with DVM. To be fair, the two predictive models adopted same feature extraction method. Here, Gaussian function was chosen by SVM as the kernel function. A general grid search method was employed to optimize SVM's two parameters (kernel width parameter *γ*, regularization parameter *C*) and they were tuned to *γ*=0.01 and *C*=0.6 respectively.

**Figure 3:**
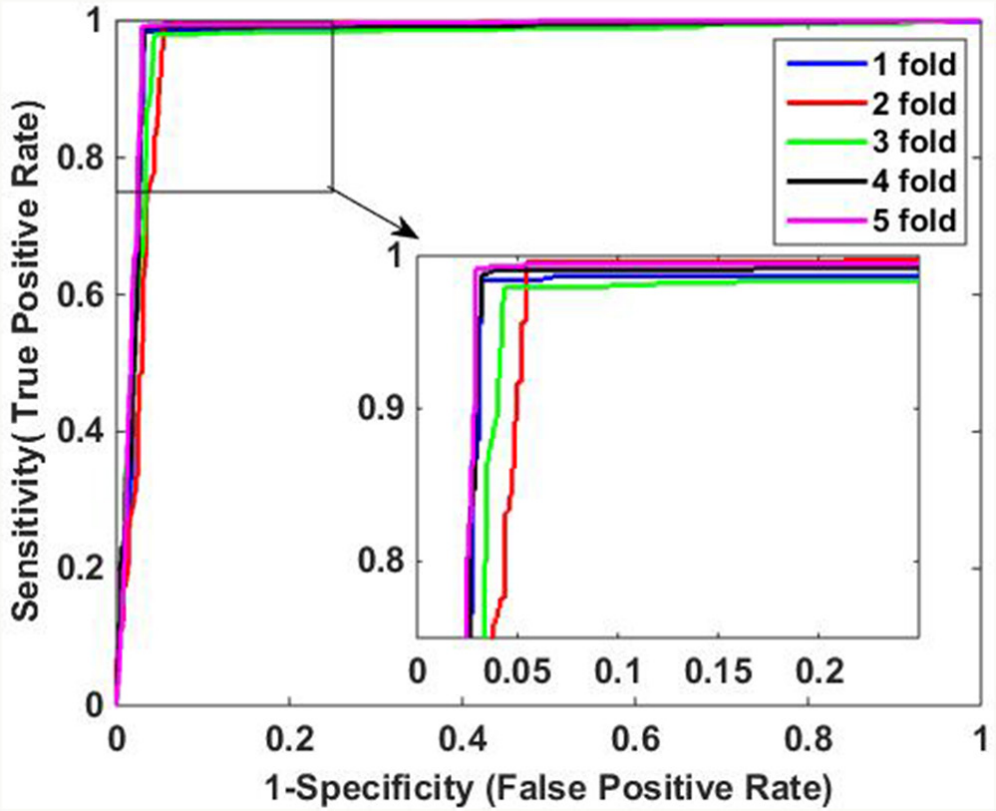
ROC curves of proposed DVM-based method on Human data set

**Figure 4:**
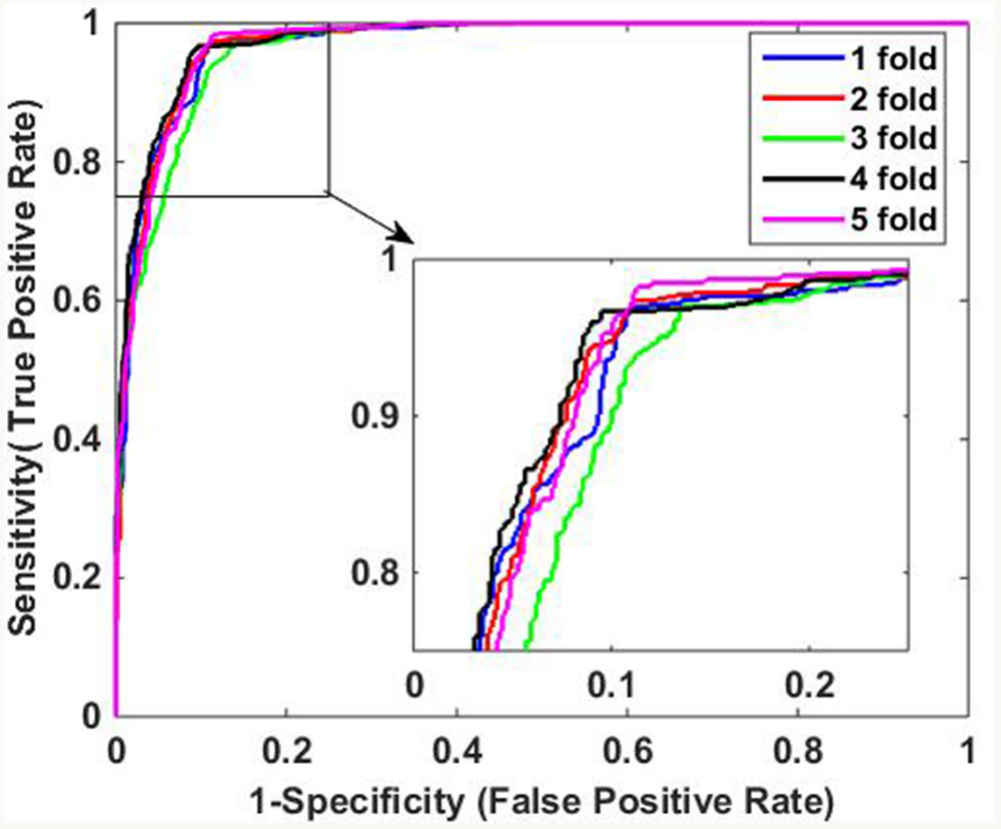
ROC curves of SVM-based method on Human data set

The predictive results of the two methods are illustrated in Table 3. When using DVM classifier to identify the PPIs on *Human* data set, we got promising results with average accuracy, sensitivity, precision and MCC of 97.30%, 95.70%, 98.61% and 94.63%, respectively. Meanwhile, SVM-based method had relatively poor performance with lower average accuracy, sensitivity, precision and MCC of 90.60%, 91.61%, 89.01% and 81.22%, which indicate that DVM has better performance than SVM for predicting PPIs. In addition, it can be observed that DVM is more stable than SVM because the former has lower standard deviations of evaluation criteria than the latter. Specifically, DVM-based method yielded standard deviations of accuracy, sensitivity, precision and MCC as low as 0.60%, 0.87%, 0.80% and 1.20%, which is less than the corresponding values of 0.95%, 0.89%, 1.72% and 1.82% of SVM-based method. Furthermore, Figure 3 and Figure 4 show the ROC curves performed by DVM and SVM, respectively. It can be observed that DVM yielded higher average AUC (area under an ROC curve) value than that of SVM classifier.

**Table 3:** Five-fold cross validation results performed on Human data set

Model	Test set	Acc (%)	Sen (%)	Pre (%)	MCC (%)
DVM	**1**	97.18	95.61	98.40	94.37
	**2**	97.30	95.05	99.62	94.71
	**3**	96.38	94.73	97.43	92.75
	**4**	97.73	96.29	98.95	95.48
	**5**	97.92	96.83	98.65	95.82
	**Average**	**97.30±0.60**	**95.70±0.87**	**98.61±0.80**	**94.63±1.20**
SVM	**1**	89.89	90.83	88.21	79.79
	**2**	91.54	91.79	91.57	83.08
	**3**	89.40	90.78	86.99	78.82
	**4**	90.93	92.96	88.64	81.95
	**5**	91.24	91.68	89.66	82.44
	**Average**	**90.60±0.95**	**91.61±0.89**	**89.01±1.72**	**81.22±1.82**

By analyzing the experimental results, we can conclude that DVM is more effective and robust than SVM in predicting PPIs. There are two possible explanations for the results. (1) Based on *k* nearest neighbors (kNNs), the robust M-estimator and manifold regularization, DVM decreases the influence of outliers and overcomes the shortcoming of the kernel function required to satisfy the Mercer condition. (2) Although there are three parameters (*β*, γ, and *θ*) to be tuned in DVM, those parameters slightly affect the performance of DVM if they are adjusted in suitable ranges. Therefore, DVM is more suitable for predicting PPIs than SVM.

### Performance on independent data set

Although our proposed method had achieved good performance for PPIs prediction on *Yeast*, *H. pylori* and *Human* data sets, we still carried out extensive analyses to verify its ability for predicting PPIs from other species (*E. coli, C. elegans*, *H. sapien*, *H. pylori* and *M. musculus*). In the following experiments, we used 11188 samples of *Yeast* data set for training and samples from other five species for testing. The corresponding feature extraction method is same to the previous experiments. The predictive results are listed in Table 4. The basis of this hypothesis is that homologs tend to be similar functional behavior and so they preserve the same PPI [18]. When applying the proposed method to the prediction of PPIs from these five species, the average accuracies of them vary from 76.23 to 92.72. On the one hand, these promising results obtained indicate that *Yeast* protein may have a similar interacting mechanism with other five species and its sequence data is sufficient for the prediction of PPIs from other species; on the other hand, it demonstrates the proposed method has good generalization ability. In addition, the prediction results fully demonstrate that it is possible that PPIs in one species can be employed to identify PPIs in other species.

**Table 4:** Predictive results of proposed method on five other species

Species	Test pairs	Accuracy
***E. coli***	6954	76.23%
***C.elegans***	4013	92.72%
***H.sapien***	1406	89.40%
***H. pylori***	1420	86.37%
***M.musculus***	312	87.69%

### Comparison with other methods

So far, a variety of machine-learning based computational methods have been proposed for PPIs prediction. To further validate the effectiveness of our method, we also compared our DVM-based predictive model using IWLD descriptor with several other previous methods (see Table 5 and Table 6) on *Yeast* and *H. pylori* data sets. In Table 5, the prediction accuracy of other previous methods on *Yeast* data set varies from 75.08% to 93.92%, while our proposed method achieves higher value of 96.52%. Similarly, for sensitivity and precision, our predictive model yields better performance than the others. Moreover, the corresponding standard deviations indicate the proposed method is stable and robust. Considering ensemble classifier usually has better performance than single classifier, although RF + PR-LPQ method has lower standard deviations, our method can also be viewed as one of the most competitive computational methods for predicting PPIs.

**Table 5:** Predictive results of different methods on Yeast data set

Model	Test set	Acc (%)	Sen (%)	Pre (%)	MCC (%)
**Guo [20]**	ACC	89.33±2.67	89.93±3.68	88.87±6.16	N/A
	AC	87.36±1.38	87.30±4.68	87.82±4.33	N/A
**Yang [21]**	Cod1	75.08±1.13	75.81±1.20	74.75±1.23	N/A
	Cod2	80.04±1.06	76.77±0.69	82.17±1.35	N/A
	Cod3	80.41±0.47	78.14±0.90	81.66±0.99	N/A
	Cod4	86.15±1.17	81.03±1.74	90.24±1.34	N/A
**You [22]**	EELM	87.00±0.29	86.15±0.43	87.59±0.32	77.36±0.44
**Wong [23]**	RF+PR-LPQ	93.92±0.36	91.10±0.31	96.45±0.45	88.56±0.63
**Our method**	DVM	**96.52±0.46**	**94.86±0.59**	**98.11±0.48**	**93.08±0.92**

**Table 6:** Predictive results of different methods on *H. Pylori* data set

Model	Acc (%)	Sen (%)	Pre (%)	MCC (%)
**Nanni *et al*. [24]**	83.00	86.00	85.10	N/A
**Nanni *et al*. [25]**	84.00	86.00	84.00	N/A
**Nanni *et al*. [26]**	86.60	86.70	85.00	N/A
**You *et al*. [22]**	87.50	88.95	86.15	78.13
**Martin *et al*. [27]**	83.40	79.90	85.70	N/A
**Wong *et al*. [23]**	89.47	89.18	89.63	81.00
**Our method**	**91.80**	**92.15**	**91.47**	**83.60**

The similar results of different methods on *H. pylori* data set can also be found in Table 6. The accuracies of other methods vary from 83.00% to 89.47% while our proposed method attains relatively higher value of 91.80%. The same is true for precision, sensitivity and MCC. The predictive results in Table 5 and Table 6 indicate that the DVM-based classifier incorporating IWLD descriptor can improve the performance of PPIs compared with the state-of-the-art methods. The promising prediction results of our method may contribute to the novel feature extraction method which can provide highly discriminative information, and the selection of DVM classifier which has been demonstrated to be robust and powerful [19].

## CONCLUSIONS

In this work, we put forward a novel evolutionary information based computational model for predicting PPIs, which combines our newly developed discriminative vector machine classifier (DVM) and an improved Weber local descriptor (IWLD) to capture highly discriminative information. To minimize data dependence and avoid the over-fitting, five-fold cross-validation was adopted accordingly. When applied to *Yeast* and *H.Pylori* data sets, the model achieves promising prediction accuracies of 96.52% and 91.80%, respectively. Additionally, to evaluate the generalization capability of the proposed method, extensive experiments are performed to predict the PPIs on five other species data sets. Besides, it is compared with SVM-based model and other previous works. The achieved results show that the proposed method is very competitive for predicting PPIs and can be taken as a useful supplementary tool to the traditional experimental methods for future proteomics research.

## MATERIALS AND METHODS

### Golden standard data sets

In this study, we verified the proposed method on a high-confidence PPIs data set *Yeast*, gathered from the publicly available database of interaction proteins (DIP), version DIP_20070219 [4]. All protein pairs were aligned by a multiple sequence alignment tool, CD-HIT [28]. To reduce fragments and similarity, those protein pairs with ≤ 50 residues or ≥40% sequence identity were all removed. Then the remaining 5594 interacting protein pairs form the positive data set and 5594 additional protein pairs from different subcellular localizations were chosen to construct the negative data set. Therefore, the data set of *Yeast* finally contains 11188 protein pairs of which half are positive samples and half negative samples.

To further test the generality of the proposed method, we also evaluate it on two other PPIs data sets: *Human* and *H. pylori*. The first data set *Human* comes from the human protein references database (HPRD). By using the aforementioned steps, we selected 3899 protein pairs as the positive data set and 4262 additional protein pairs from different subcellular localizations as negative data set. As a result, the *Human* data set finally consists of 8161 protein pairs. Similarly, the second data set *H. pylori* consists of 2916 protein pairs, of which half are interacting pairs and half non-interacting pairs, as described by Martin *et al*.

### Improved Weber local descriptor

**Figure 5:**
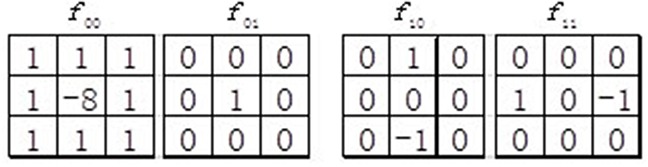
Four filters used in the original WLD

Inspired by Weber's Law, Chen *et al*. [29] proposed the original Weber local descriptor (WLD) for image recognition, which contains two components, namely differential excitation and orientation. Differential excitation component *ξ*(x_i_) of WLD is the ratio between two terms: One is the relative intensity differences of an interest point *x_i_* against its neighbors; the other is the intensity of *x_i_* itself. We first calculate the intensity differences between *x_i_* and its neighbors with the filter *f*_00_ (see Figure 5):

$$v_{i}^{00}=\sum_{j=0}^{p-1}\left(x_{j}-x_{i}\right)$$ (1)

where *x_j_* (*j* = 0,1,.., *p* − 1) represents the *jth* neighbor of *x_i_* and *p* is the number of its neighbors. We then calculate the ratio of the intensity differences $v_{i}^{00}$ and $v_{i}^{01}$:

$$G_{r}\left(x_{i}\right)=\frac{v_{i}^{00}}{v_{i}^{01}}$$ (2)

where $v_{i}^{01}$ is the output of the filter *f*_01_ (see Figure 5). As described before, $v_{i}^{01}$ is just the original intensity of *x_i_*. Next, the arctangent function is employed to construct the differential excitation $\xi \left(x_{i}\right)(\in [-\pi /2,\pi /2])$:

$$\begin{array}{c} \xi \left(x_{i}\right)=\text{arctan}\left(G_{r}\left(x_{i}\right)\right)=\text{arctan}\left(\frac{v_{i}^{00}}{v_{i}^{01}}\right)\\ =\text{arctan}(\overset{p-1}{\underset{j=0}{\sum }}\left(\frac{x_{j}-x_{i}}{x_{i}}\right)) \end{array}$$ (3)

**Figure 6:**
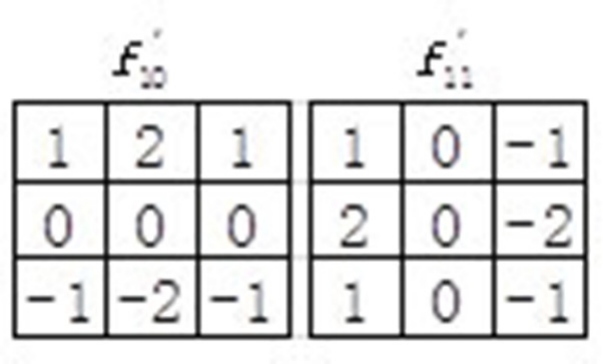
Sobel operators used in the improved WLD (IWLD)

In addition, orientation component of WLD describes the gradient orientation of interest point. In the original WLD, only 4 neighbors of *x_i_* are utilized which may lose some important discriminating information and are sensitive to noise. In our study, we adopted an improved WLD (IWLD) descriptor by introducing Sobel operators (see Figure 6). By taking into account all 8 neighbors of *x_i_*, it can not only preserve sufficient orientation information but also effectively suppress the noise. Thus, the orientation component of IWLD $\gamma \left(x_{i}\right)$ is computed as:

$$\gamma \left(x_{i}\right)=\text{arctan}2\left(\frac{v_{i}^{11}}{v_{i}^{10}}\right)+\pi$$ (4)

where $v_{i}^{10}$ and $v_{i}^{11}$ denote the outputs of the filters $f_{10}^{'}$ and $f_{11}^{'}$ (see Figure 6).

To perform histogram statistics, the differential excitation *ξ*(x_i_) is quantized into *M* intervals $l_{m}(l_{m}=\left[\eta_{m}^{l},\;\eta_{m}^{u}\right],m=0,2,\ldots ,M-1),$ where $\eta_{m}^{l}=\left(\frac{m}{M}-1/2\right)\pi$ is the lower bound and $\eta_{m}^{u}=\left(\frac{m+1}{M}-1/2\right)\pi$ is the upper bound. So, the value of *m* is calculated as follow:

$$m=mod(\left[\frac{\xi \left(x_{i}\right)+\frac{\pi }{2}}{\frac{\pi }{M}}\right],M)$$ (5)

Similarly, $\gamma \left(x_{i}\right)(\in (0,2\pi ))$ is also quantized into *T* dominant orientations as follow:

$$\Phi_{t}=f_{q}\left(\gamma \right)=\frac{2t}{T}\pi \text{ and }t=mod(\left[\frac{\gamma \left(x_{i}\right)}{2\pi /T}+\frac{1}{2}\right],T)$$ (6)

By calculating *m*, *t* value of each point in an image, a 1D histogram vector $S=\text{\{}s_{m,t}\text{\}}$  (m=0,1,…,M−1, t=0,1,…, T−1) can be obtained accordingly. To fully mine the local discriminative information, we first divide the image into *V* × *H* sub blocks. Here, *V* represents the number of sub blocks in vertical direction and *H* represents the number of sub blocks in horizontal direction, and the histogram vector of each block is obtained accordingly. Then all the histogram vectors of the image are concatenated into the final one-dimensional IWLD feature vector.

In this work, there are four free parameters (*M, T, V, H*) to be tuned. Through grid search on *Yeast* and *H. pylori* data sets, we chose *M*=8, *T*=8, *V*=*H*=2 in our experiments and each protein sequence sample is transformed into a 256 dimensional IWLD vector. Next, every two IWLD vectors from corresponding protein pairs are concatenated into a 512 dimensional vector. Then, the dimensionality reduction algorithm PCA is employed to reduce the impact of noises and accelerate the predictive process, and the final 200 dimensional reduced vector is constructed for the subsequent classification.

### Discriminative vector machine

Classification is a fundamental issue in pattern recognition field and there exist numerous classification algorithms for different recognition tasks. In this work, our newly developed discriminative vector machine (DVM) classifier [19] was adopted in classification. DVM is a probably approximately correct (PAC) learning classifier which can reduce the error caused by generalization and is very robust. For a given test sample *y*, the first step of DVM is to find its *k* nearest neighbors (kNNs) to suppress the effect of outliers. The kNNs of *y* can be expressed as $\;X_{k}=[x_{1},x_{2},\ldots ,x_{k}]$, where *x_i_* denotes the *ith* nearest neighbor. Equally, *X_k_* can also be represented as $\;X_{k}=[x_{k,1},x_{k,2},\ldots ,x_{k,c}],$, where *x_k,j_* comes from the *jth* class. So the objective of DVM is to solve the following minimization problem:

 βkminδ||β||k+∑i=1d∅((y−Xkβk)i)+γ∑p=1k∑q=1kwpq(βkp−βkq)2 (7)

where $\beta_{k}$ can be denoted as $\left[\beta_{k}^{1},\beta_{k}^{2},\ldots ,\beta_{k}^{k}\right]$ or $[\beta_{k,1},\beta_{k,2},\ldots ,\beta_{k,c}],$ where $\beta_{k,i}$ is the coefficient from the *ith* class, $\left||\beta_{k}|\right|$ is a norm of $\beta_{k}$ and the corresponding *L*_2_ norm is employed in our calculation, ${\left(y-X_{k}\beta_{k}\right)}_{i}\;$ is the *ith* element of $y-X_{k}\beta_{k}$ and ∅ is a robust M-estimator to improve the robustness of DVM. M-estimator is a generalized maximum likelihood operator proposed by Huber to estimate parameters under the cost function [30]. In this work, a robust Welsch M-estimator $\left(\emptyset \left(x\right)=(1/2)(1-exp(-x^{2})\right)$ is adopted to attenuate error so that outliers would have a less impact on classification. The last section of Eq. (7) is a manifold regularization where $w_{pq}$ is the similarity between the *pth* and the *qth* nearest neighbors of *y*. In this work, $w_{pq}$ is defined as the cosine distance between the *pth* and the *qth* NN of*y*. Then the corresponding Laplacian matrix *L* can be expressed as

L=D−W (8)

where *W* is the similarity matrix whose element is $\;w_{pq}\left(p=1,2,\ldots ,k;q=1,2,\ldots ,k\right),$, *D* is a diagonal matrix whose *ith* element *d_i_* is the sum of $w_{iq}(q=1,2,\ldots ,k)$. According to Eq. (8), the last section of Eq. (7) can be rewritten as $\;\gamma \beta_{k}^{T}L\beta_{k}.$ Furthermore, a diagonal matrix $P=\text{diag}(p_{i})\;$ is constructed and its element $p_{i}\left(i=1,2,\ldots ,d\right)\;$ is denoted as:

$$p_{i}=e^{-\frac{{\left({\left(y-X_{k}\beta_{k}\right)}_{i}\right)}^{2}}{\sigma^{2}}}$$ (9)

where *σ* is the kernel size which can be calculated in the following form:

$$\sigma =\sqrt{(\theta *{\left(y-X_{k}\beta_{k}\right)}^{T}*(y-X_{k}\beta_{k})/d}$$ (10)

where *d* is the dimension of *y* and *θ* is a constant to curb outliers. In this work, it is assigned to 1.0 as in the literature [31]. By merging Eq. (8), (9) and (10), the minimization of Eq. (7) can be converted to the following problem:

arg(y−Xkβk)βkminTP(y−Xkβk)+δ∥βk∥22+γβkTLβk (11)

According to the theory of half-quadratic minimization, the global solution $\beta_{k}$ can be described as:

$$\beta_{k}={\left(X_{k}^{T}PX_{k}+\delta I+\gamma L\right)}^{-1}X_{k}^{T}Py$$ (12)

After the related coefficients are calculated, the test sample *y* can be identified as the *ith* class if the residual $y-X_{ki}\beta_{ki}$ is the minimum value.

Ri=∥i   miny−Xkiβki∥,  i=1,2,…,c (13)

By means of robust M-estimator and manifold regularization to suppress the effect of outliers and strengthen its discriminatory ability, DVM classifier has better robustness and higher generalization ability than kNNs. In this work, there are two classes in total to be identified: non-interacting protein pair (class 1) and interacting pair (class 1). If the residual *R*_1_ is the minimum distance, the test sample *y* would be classified as non-interacting protein pair, or it would be identified as interacting protein pair. For three free parameters (*δ*, *γ*, *θ*) of DVM model, it is time-consuming to directly search for their optimal values. It is gratifying that DVM algorithm is so stable that all these parameters only affect the performance slightly if they are set in feasible ranges. Based on above knowledge and through grid search, the parameters *δ* and *γ* are set as 1E-3 and 1E-4 respectively. Just as described before, *θ* is a constant and is always set to 1 throughout the entire process. For large data set, DVM classifier needs to spend relatively more time in finding the representative vector, so multi-dimensional indexing techniques can be adopted to speed up the search process to a certain extent.

### Procedure of proposed model

**Figure 7:**
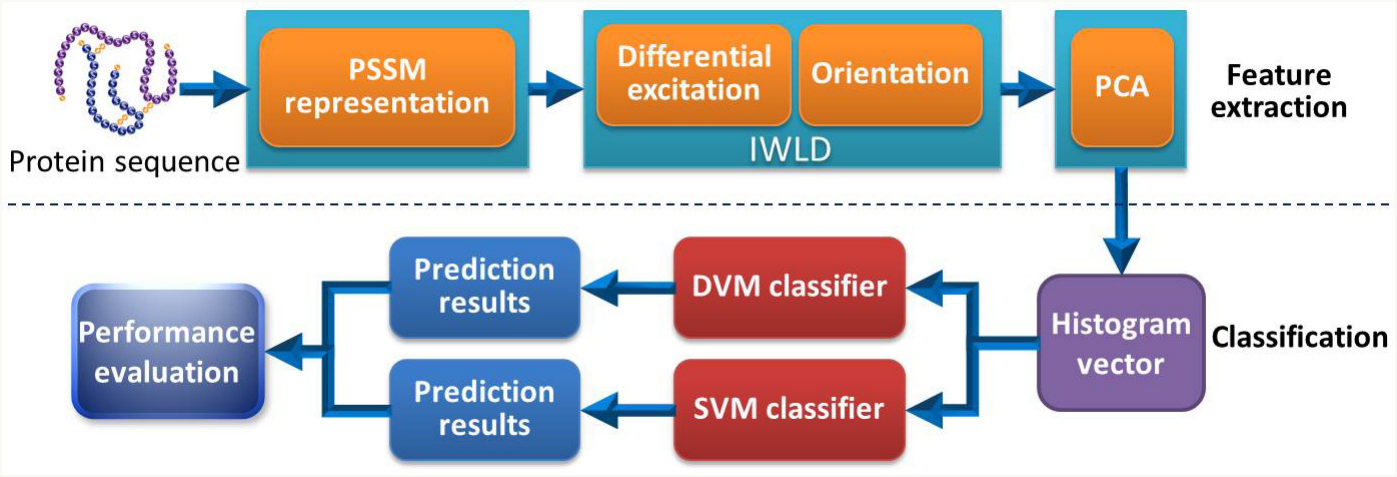
Flow chart of our proposed method for the prediction of PPIs

The procedure of our proposed model mainly contains two steps: feature extraction and classification. The feature extraction is also divided into three steps: (1) the PSI-BLAST tool is used to represent each protein sequence and PSSM is obtained accordingly; (2) The PSSM from each protein is transformed into the corresponding histogram vector via IWLD descriptor; (3) Dimensional reduction of the histogram vector is performed by PCA algorithm. In the same way, sample classification also consists of two steps. (1) Based on the data sets of *Yeast*, *H. pylori* and *Human*, DVM model is trained and used to carry out classification; (2) The trained DVM model is then employed to predict the PPIs and its performance is evaluated accordingly. Furthermore, SVM model is also constructed for predicting PPIs on *Human* data set and the corresponding evaluation is also performed. The overall flow chart of our method is shown in Figure 7.

### Evaluation criteria

To evaluate the performance of related predictive methods, four criteria, including the accuracy (*Acc*), sensitivity (*Sen*), precision (*Pre*), and Matthews's correlation coefficient (*MCC*), were introduced, which can be calculated as follows:

$$Acc=\frac{TP+TN}{TP+FP+TN+FN}$$ (1)

$$Pre=\frac{TP}{TP+FP}$$ (2)

$$Sensitivity=\frac{TP}{TP+FN}$$ (3)

$$MCC=\frac{\left(TP\times TN)-(FP\times FN\right)}{\sqrt{\left(TP+FN)\times (TN+FP)\times (TP+FP)\times (TN+FN\right)}}$$ (4)

where *TP* (true positive) represents the number of interacting protein pairs predicted correctly while *FP* (false positive) denotes the number of non-interacting protein pairs predicted falsely. Similarly, *TN* (true negative) stands for the number of non-interacting protein pairs predicted correctly, and *FN* (false negative) denotes the number of interacting protein pairs predicted falsely. Receiver-operating characteristics (ROC) curve is a standard technique for summarizing classifier performance over a range of trade-offs between TP and FP error rates. In our study, ROC curves were also calculated to evaluate the validity of prediction models.
